# Editorial: Functional analysis of species-specific non-coding RNAs in plants

**DOI:** 10.3389/fgene.2022.1105433

**Published:** 2023-01-04

**Authors:** Menglei Wang, Yue Xiao, Nan Su, Yuepeng Song

**Affiliations:** ^1^ National Engineering Research Center of Tree Breeding and Ecological Restoration, College of Biological Sciences and Technology, Beijing Forestry University, Beijing, China; ^2^ Key Laboratory of Genetics and Breeding in Forest Trees and Ornamental Plants, Ministry of Education, College of Biological Sciences and Technology, Beijing Forestry University, Beijing, China

**Keywords:** miRNA, non-coding RNA, developmental plasticity, abiotic stress, biotic stress

## Introduction

In addition to protein-coding RNAs, eukaryotes have different types of non-coding RNAs (ncRNAs) that are involved in the regulation of complex molecular and cellular processes. The sequences and functions of these ncRNAs are species-specific. Research on ncRNAs in plants has flourished during the past decade, due to advances in high-throughput sequencing (HTS) technologies and pioneering studies that have revealed the high frequency of ncRNAs in plants. NcRNAs can be divided into small RNAs, medium ncRNAs, and lncRNAs, among which small RNAs, particularly microRNAs (miRNA) and lncRNAs, play important roles in plant growth and development, critical phase transition, developmental plasticity, and response to biotic and abiotic stresses. Studying ncRNAs and their functions in a wider range of species will help elucidate the evolutionary pathway of ncRNA-mediated regulation mechanisms in plants and provide a theoretical basis for the study of genes that are constantly updated in response to changes in the natural environment. In this Research Topic, we present the latest advances in research of ncRNA regulation in plants, including their regulatory roles in plant developmental plasticity and abiotic and biotic stress responses. To date, ncRNAs have been studied in 179 species (Guo et al., 2022), among which this Research Topic covers 12, three-quarters of which are newly discussed, including the rare wood species *Dalbergia odorifera* and the low-temperature-tolerant *Ammopiptanthus nanus*. The findings reported in these studies provide valuable information toward the further identification of ncRNA functions and targets in a wider range of species.

### Known miRNA regulatory network conserved in new species

MiRNA is an endogenous small ncRNA with important regulatory functions in eukaryotes (Reinhart et al., 2000; Bartel, 2004). Plant miRNA mainly recognizes and degrades target genes or represses the translation of target genes by recognizing complementary sequences at the post-transcriptional level, thereby participating in the regulation of plant growth and developmental plasticity (Song et al., 2019). Conserved miRNA emerged in the early stages of plant evolution, compared to other miRNA families, and they have high expression abundance and function conservation features. MiRNAs typically target multiple members of a gene family due to their mechanism of action, and conserved miRNA tends to target conserved genes with similar functions (Bartel, 2004; Song et al., 2019).

In a study of newly discovered ncRNAs in species such as *Dalbergia odorifera* and *Ammopiptanthus nanus*, 302 conserved miRNAs and 282 novel miRNAs belonging to 79 conserved miRNA families were found, among which the expression patterns and functions of the target genes of conserved miRNAs were consistent with those reported in model plants. These findings suggest that functional conserved miRNAs that emerged in the early evolution of species are functionally conserved in different plant groups, thus determining their critical roles in plant morphological differentiation, developmental plasticity, and environmental stress responses (Table 1).

**TABLE 1:** Conserved and species-specific miRNA of new species in this Research Topic.

Species	Conserved and species-specific miRNA	Target genes	Function
*Betula luminifera*	miR156/miR164/miR166	*SPL13/NAC100/HHP1*	Low nitrogen stress
*Dalbergia odorifera*	miR156	*SPL6, SPL12*	Xylem differentiation
*Moso bamboo*	miR166	*PeHOX10, PeHOX32*	Vascular tissue differentiation
*Vaccinium corymbosum*	miR166	*HD-ZIP III*	Freezing, cold, heat, salt stress
*Sonneratia apetala*	miR396	*UBP*	Salt stress
*Ammopiptanthus nanus*	miR4415	*L-AO*	Cold acclimation

The full names of the abbreviations in the table are as follows, *SPL: Squamosa promoter-binding protein-like, HHP1: Heptahelical protein 1, HD-ZIP III: Homeobox-leucine zipper proteins, UBP: Ubiquitin-specific protease, L-AO: L-ascorbate oxidase gene*.

### Species-specific ncRNAs in plant developmental plasticity

LncRNAs are RNAs that are >200 bp in length and lack open reading frames (ORFs) or protein-coding capacity. They have higher tissue specificity, lower expression, and lower sequence conservation among species compared to mRNA in plants (Bardou et al., 2014). Unlike miRNA, lncRNA affects the transcription efficiency of neighboring genes while also forming scaffolds and decoys, or encoding small peptides. Recently, with the continuous development of sequencing technology, lncRNAs have been identified in increasing numbers of plant species in various growth stages, indicating their essential roles in modulating diverse biological regulatory processes in plants (Bartel, 2004; Chekanova, 2015; Chekanova, 2021).

LncRNAs participate in poplar lignin biosynthesis with the involvement of transcription factors and miRNAs. Zhang et al. (2022) reported that differentially expressed woody plant lncRNAs and target genes in two poplar genotypes were directly coexpressed with MYB and VND transcription factors and structural genes in the lignin and flavonoid pathways. Numerous auxin- and gibberellin-related lncRNA-mRNA coexpression networks have also been identified; these may regulate secondary xylem during the formation of tension wood. Together, these results suggest that lncRNAs are widely involved in lignin and flavonoid metabolism in poplars, complementing recent findings on new ncRNA members and their regulatory pathways, and providing a theoretical basis for exploring the function of ncRNAs in the developmental plasticity of plants.

### Species-specific miRNAs and lncRNAs in abiotic stress responses in plants

In addition to conserved miRNAs, which have mainly analogical functions among plant species, some species also have non-conserved, species-specific miRNAs. Species-specific miRNAs typically have unique functional roles, and their existence provides clues that can be used to study the functions of corresponding *MIR* genes to understand the evolutionary positions of these genes (Song et al., 2019; Yu et al., 2019).

The leguminous plant *Ammopiptanthus nanus* has excellent tolerance to low temperature, and has therefore been used to study the molecular mechanisms of plant responses to low-temperature stress. The legume-specific miRNA miR4415 is involved in cold acclimation in *A*. *nanus* by targeting an L-ascorbate oxidase gene that regulates apoplast redox status. This research has provided a basis for investigating cold acclimation regulation by miRNA.

**FIGURE 1:**
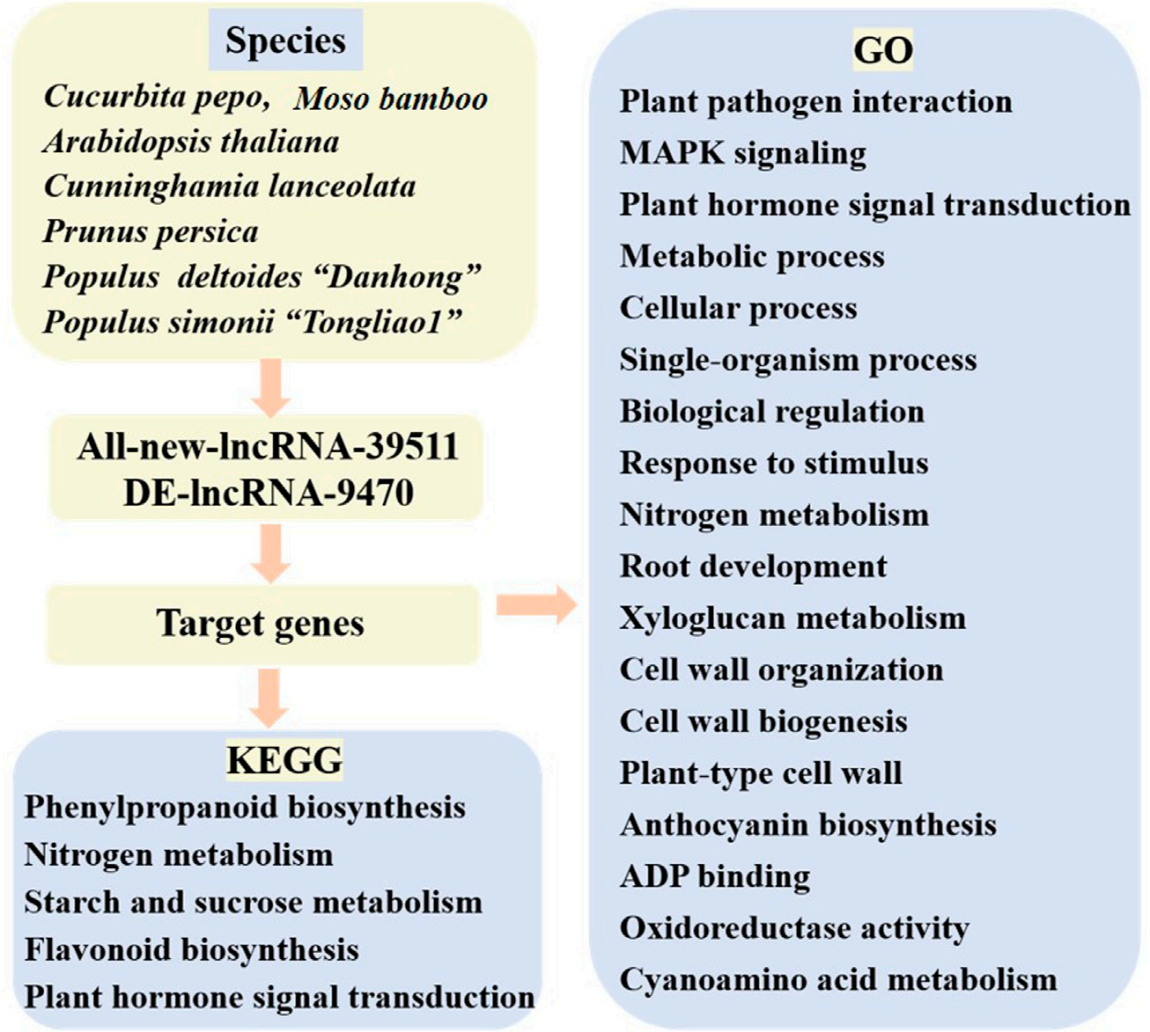
Novel lncRNA and their regulatory function of target genes in this issue.

In this Research Topic, we present ncRNA studies on *Cucurbita pepo*, *Moso bamboo*, *Arabidopsis thaliana*, *Cunninghamia lanceolata*, *Prunus persica*, *Populus deltoides* “Danhong”, and *Populus simonii* “Tongliao1” that have reported 39,511 lncRNAs, among which most are novel. Together, these studies detected 9,470 differentially expressed lncRNAs showing targeted regulation of genes involved in processes related to powdery mildew, nitrogen metabolism, compression stress, ultraviolet B-induced flesh anthocyanin biosynthesis, wood formation, as well as phenylalanine molecular pathways and other biological processes (Figure 1).

In addition to developmental plasticity in plants, lncRNAs are widely involved in abiotic and biotic stress responses. The stress responses of lncRNAs differ from those of miRNAs. LncRNAs differentially expressed under drought, cold, salt, heat, and abscisic acid stress have been well described in model plants such as *Arabidopsis thaliana* and rice (Bartel, 2004; Zhang et al., 2022).

Light is a critical factor influencing anthocyanin biosynthesis in plants depending on light intensity, duration, and other light qualities. A recent genome-wide study based on transcriptomes of the flesh and juice of peach (*Prunus persica*) fruits at different developmental stages to identify lncRNAs involved in fruit ripening identified differentially expressed lncRNAs including XLOC_011933, XLOC_001865, and XLOC_042291. These are involved in ultraviolet B-induced anthocyanin biosynthesis and positively regulate *UVR8* and *COP10*, which participate in anthocyanin biosynthesis in peach fruits (Zhang et al., 2022).

LncRNAs are also involved in compression stress. In *Cunninghamia lanceolata*, lncRNA transcript_31,838 and transcript_29,184 are significantly correlated with *CESA*.*2*, *CESA2*.*2*, *CESA4*, *MANS*.*1*, *MYB21*, *MYB128*, *PAL1*, and *CCoAOMT1*, and may target these genes through transregulation in response to compression stress, thereby regulating the production of compression wood.

### Interactions between miRNAs and lncRNAs in response to stress in plants

LncRNAs also act as precursors to small RNAs and interact with miRNAs in a process known as target mimicry to regulate miRNA activity and abundance. This mechanism has also been linked to plant responses to abiotic stress. In nitric oxide-treated *rhd6* and wild-type *A*. *thaliana*, *MIR5658* and *MIR171* precursors are highly upregulated, and together with the novel lncRNAs MSTRG 15935, 15936, and 17,591, interact with differentially expressed protein coding genes involved in hormone signaling, cell wall development, and root hair formation. This makes them candidate genes for cell wall regulation and root hair phenotype recovery under nitric oxide treatment.

RNA sequencing analyses of the expression profiles of mRNAs, miRNAs, and lncRNAs in bamboo roots under different nitrogen treatment levels demonstrated a nitrogen metabolism regulatory network, which included 17 nitrogen metabolic pathway genes, 4 miRNAs targeting three *NPF* genes, and 10 lncRNAs targeting *NPFs* and *GDHs* through 15 transcription factors, indicating an ncRNA nitrogen metabolism regulation mechanism in *Moso bamboo*. Collectively, these findings demonstrate that lncRNA interactions with miRNA are essential in plant responses to abiotic stress.

In response to pathogen attack, plant cells trigger downstream molecular signaling networks. Pathogens such as powdery mildew, stripe rust, and rice blast often reduce the production of economic crops such as wheat and rice. Previous studies have reported the roles of ncRNAs in plant responses to such biological stresses. In one study, overexpression of the lncRNA ALEX1 in rice led to jasmonic acid pathway activation and resistance to bacterial blight (Yu et al., 2020). In another study on *Vitis vinifera*, 71 lncRNAs responsive to powdery mildew and 83 responsive to downy mildew were identified based on transcriptome sequencing responses to these obligate biotrophic fungal phytopathogens (Bhatia et al., 2021). Tian et al. inoculated powdery mildew into *Cucurbita pepo* leaves, and identified 242 differentially expressed lncRNAs. Genome-wide profile analysis predicted interactions between these lncRNAs and miRNAs as well as target genes associated with plant-pathogen interactions, MAPK signaling, and plant hormone signal transduction pathways. These findings suggest that *C*. *pepo* lncRNAs responsive to powdery mildew may participate in the pathogen response by regulating the expression of genes related to plant-pathogen interactions.

RNA interference (RNAi) technology, a form of post-transcriptional gene silencing induced by double-stranded RNA, has recently been applied to control plant pathogens and pests. RNAi depends on the recognition of target genes by silencing RNA (siRNA) (Fire et al., 1998). RNAi-induced gene silencing is an effective tool for enhancing pest and disease resistance in plants and genetic improvement in crops. Carrying out RNAi using a nano-carrier delivery system has been applied in biopesticide research; for example, multivariate nano-biotics have been successfully prepared to overcome the short duration and poor efficacy of plant pesticides using a nanoparticle-mediated delivery system that simultaneously loaded double-stranded RNA and plant-derived pesticides (Shen et al., 2022). This technique provides a new strategy for the development of biopesticides and opens a new chapter for the integration of ncRNA functional studies of the biological regulation of plant responses to pathogen attack and insect resistance (Li et al., 2022).

## Prospects for future ncRNA research

NcRNAs are key regulatory factors in plant growth and development and play an indispensable role in maintaining the balanced expression of functional genes. The continual discovery of new ncRNAs in different species has accelerated progress in ncRNA characterization and functional analysis. However, ncRNAs have been studied in only 1% of plant species, with a focus on model and economically important species, and many species with evolutionary significance remain to be studied. The study of ncRNAs in new species will contribute to the comprehensive analysis of important nodes in species evolution.

The rapid development of ncRNA research in recent years has been made possibly by increasingly updated HTS technology. In the future, single-cell RNA sequencing, which is far superior to the current technology in terms of sequencing throughput, and spatial RNA sequencing, which spatially resolves RNA activity while comprehensively analyzing RNA transcription, will provide new opportunities to discover new ncRNAs and further analyze the biological functions of those that are already known.

In addition to their involvement in the development and stress responses of plants, new research directions to analyze ncRNA functions such as long-distance transport, plant-microbial interactions, and nanomaterial delivery are worthy of attention and further research. Nanoparticles can be applied to carry gene-editing elements through the cell wall for genetic transformation in plants; thus, it is also worth exploring whether ncRNA functions can be studied by combining nanoparticle-mediated delivery systems with gene-editing systems.
